# Encoding Temporal Features of Skilled Movements—What, Whether and How?

**DOI:** 10.1007/978-3-319-47313-0_3

**Published:** 2016-09-29

**Authors:** Katja Kornysheva

**Affiliations:** 8 Institute of Cognitive Neuroscience, University College London, London, UK; 9 Department of Neuroscience, Erasmus MC, Rotterdam, The Netherlands; 10 School of Psychology, Bangor University, Bangor, Wales UK

**Keywords:** Motor timing, Spatiotemporal control, Sequence learning, Modular representation, Cortico-subcortical loops

## Abstract

In order to reliably produce intelligible speech or fluently play a melody on a piano, learning the precise timing of muscle activations is essential. Surprisingly, the fundamental question of how memories of complex temporal dynamics of movement are stored across the brain is still unresolved. This review outlines the constraints that determine whether and how the timing of skilled movements is represented in the central nervous system and introduces different computational and neural mechanisms that can be harnessed for temporal encoding. It concludes by proposing a schematic model of how these different mechanisms may complement and interact with each other in fast feedback loops to achieve skilled motor timing.

## Introduction (“What”)

**Fig. 1:**
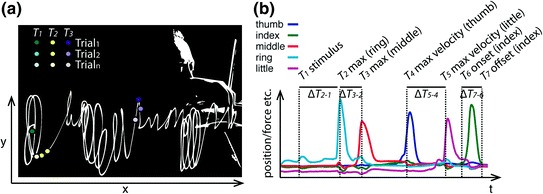
**a** Example of a skilled motor sequence depicted in two-dimensional Cartesian space (*x* and *y*) (adapted from http://www.telegraph.co.uk/culture/culturepicturegalleries/7073785/On-the-Move-Visualising-Action-at-the-Estorick-Collection-of-Modern-Italian-Art.html?image=4). Repeating the skilled sequence can lead to the clustering of time points *T*_2_ to *T*_n_ following the onset of movement (*T*_1_) respectively. Note that while here for illustrative purposes the variability of the spatial trajectory across trials is ignored, in reality the clustering across trials would take into account both space (position) and time (*colour*), cf. Laje and Buonomano (2013). **b** An example of a variable of interest during motor production such as dynamics (force) on a finger keyboard during a timed finger sequence task (adapted from Kornysheva and Diedrichsen 2014). Other variables of interest could be different kinematic measurements such as position and velocity depending on the motor task requirements. Accordingly motor timing can be quantified as time differences between task-relevant extrinsic stimuli and intrinsic states—such as maximum finger force after a go cue (Δ*T*_2–1_), eyelid position or velocity after a conditioning stimulus in eyeblink conditioning, the interval between two finger presses defined as the points of maximum velocity for each finger (Δ*T*_5–4_), or the movement duration, i.e. the difference between the offset and the onset of a movement (Δ*T*_7–6_)

In the middle of the past century, the engineer and photographer Gjon Mili developed a technique to capture trajectories of movements in space such as those produced by musicians, athletes and painters using stroboscopic cameras. He was able to record skilled movement sequences by attaching a light to the subjects’ effector of interest, such as the hand holding the violin bow, and letting the movement unfold in darkness with a long film exposure. The artist himself was only captured towards the end of the sequence when illuminating the room (Fig. 1a). Recording these trajectories revealed the skillful movement sequences humans are able to retrieve from memory and produce with their body in space. What remained invisible to Mili’s lens is how the captured trajectory unfolded in time. It is left to the observer’s imagination what velocity, acceleration and deceleration patterns the trajectory follows, how these spatial patterns emerged in time—its temporal features.

While traditionally the focus in motor neuroscience has been on the spatial dimension of movement sequences, such as the ordering or evolution of movements in space (Tanji and Shima 1994; Graybiel 1998; Hikosaka et al. 2002; Shenoy et al. 2012), the temporal dimension is equally crucial for the production of many skilled actions. Producing muscle activations in a correct order, but with inaccurate timing can have detrimental effects on performance in domains such as speech, complex tool use and music—a verbal utterance would become incomprehensible to the receiver, the tennis racket would miss the tennis ball and the violinist would desynchronize from the orchestra’s pace.

At a purely descriptive level, skilled timing of a movement sequence in space entails that the movement has a reproducible temporal structure relative to an external stimulus or an internal motor state such as the occurrence of a movement onset. Here reproducibility entails that there is a certain level of temporal accuracy—typically within tens of milliseconds for most skilled motor sequences—relative to such a point of reference, when reaching a particular extrinsically (e.g. in Cartesian space) or intrinsically (e.g. in joint or muscle space) defined state of the body. Thus, when repeating a skilled spatial sequence of movements such as the position of the hand controlling the bow, the particular points in time (*T*_2_, *T*_3_) after movement onset time (*T*_1_), cluster at the same extrinsic positions of the bow in two-dimensional space (*x* and *y* coordinates), respectively. In other words, a certain spatial configuration is reached at about the same time, with the degree of clustering reflecting the temporal precision of the movement. The temporal pattern of a movement trajectory becomes particularly evident with increased jerk, which reflects the strength of changes between acceleration and deceleration and whether the movement sequence contains activation pauses such as in a finger pressing task (Fig. 1b). Defining the motor points of interest is more straightforward for the latter type of actions (Fig. 1b), as they involve discrete kinematic events. When measuring motor timing, the timing of several kinematic and dynamic variables may be of interest depending on the motor task requirements, such as the variability of the spatial trajectory in time, the interval between an external stimulus and the maximum force, position, velocity, of a movement, etc., as well as between movements produced using the same or different effectors. Thus, in principle these variables may capture different aspects of temporal dynamics of skilled motor sequences as diverse as typing out a Morse code involving one effector and uttering a word or phrase which engages hundreds of muscles, both of which have to be executed with precise timing.

How does the nervous system represent and integrate the temporal features of such spatio-temporal sequences?

## Representation of Timing for Spatio-temporal Skills (“Whether”)

Regularity or precision of a behavioural feature such as the temporal or spatial structure of a movement does not entail that the central nervous system (CNS) forms a dedicated representation or control mechanism for this feature. While goal directed and skilled movements have been shown to be sub-served by dedicated representations of force, direction, temporal order of muscle activations or a trajectory of movement in space (Evarts 1968; Georgopoulos et al. 1982; Hikosaka et al. 2002; Averbeck et al. 2002; Churchland et al. 2006; Shima et al. 2007; Shenoy et al. 2012) the presence of a dedicated substrate for encoding the timing for spatio-temporal motor skills is under debate.

In a series of experiments, Mussa-Ivaldi and colleagues demonstrated that the motor system is inherently biased to learn velocity-dependent over time-dependent representations during force field adaptations (Conditt and Mussa-Ivaldi 1999). Subjects performed reaching movements and were perturbed by force fields dependent either on the time after movement onset (time-dependent) or on the velocity (velocity-dependent, proportional to velocity) of the movement. Crucially, aftereffects and adaptation were evaluated in the context of generalization, when subjects were tested on circular instead of the trained reaching movements. These experiments revealed that after training on a time-dependent force field, generalization to a new movement was indistinguishable from the aftereffects and adaptation to velocity-dependent training. The authors concluded that there is an automatic bias to learn state-dependent instead of time-dependent representations during motor adaptation. Notably, the force field profile employed in the time-dependent condition was designed to be similar to a velocity-dependent force field, involving a bell-shaped perturbation with a maximum force in the middle of the movement when subjects produced the highest velocity. The primacy of state-dependent representations occurred when a perturbation environment was similar to a viscous field (water like environment). It is thus feasible that time-dependent force field profiles that are less correlated with movement velocity may override this bias.

However, in a follow-up study, Mussa-Ivaldi and colleagues (Karniel and Mussa-Ivaldi 2003) demonstrate that a time-dependent force field that is uncorrelated to movement velocity still produces no motor adaptation. Here the time-dependent force followed a sinusoidal amplitude at 3 Hz and was presented continuously during the experiment. This important study suggests that the CNS is unable to form a representation of a regular, temporally predictable force profile that is uncoupled from state-dependent representation. However, the employed time-dependent perturbation was not coupled to the onset of the movement as in the previous experiment (Conditt and Mussa-Ivaldi 1999), or at least to an external cue relevant to movement initiation. It can thus be hypothesized that this link may be a constraint for the acquisition of a time-dependent movement adaptation.

Indeed, Medina and colleagues demonstrated that learning motor timing during adaptation in smooth pursuit eye movements could be independent of state-dependent encoding (Medina et al. 2005). In training trials, a target moved horizontally for a fixed duration (500 ms) and deflected vertically from a horizontal to vertical movement. Probe trials were used to assess adaptation by looking at eye movement velocity into the vertical direction. Learning to time movements correctly was independent of the position of the eyes on the horizontal plane and of the distance/velocity of the movements. Importantly the adaptation effects were dependent on the predictive power of each variable. If both the time from target motion onset and the distance travelled were equally predictive, the adapted eye movements were a mixture of the two representations, whereas if only one variable was predictive of the vertical perturbation, the adaptation reflected the learning of time or distance only, respectively. This highlights the flexibility of motor adaptation with regard to the representation of time and space depending on which variable leads to task success.

Diedrichsen and colleagues showed that time- and state-dependent representation of spatio-temporal movements that involves the coordination of two effectors—the arm and the thumb—depends on whether their activation overlaps in time (Diedrichsen et al. 2007). Following a training phase in which the movements had to be timed precisely, the subjects were asked to reduce the speed of the arm movement. The thumb press was also timed and scaled in length proportionally to the arm movements, suggesting that the thumb movement was made dependent on the state (velocity) of the arm movement and not on absolute time since arm movement onset. Interestingly, absolute timing was employed when the movements were separated in time during training, that is when the thumb preceded the arm movement by 100–500 ms. This suggests that training temporally overlapping movements produces a bias to encode the movements of multiple effectors relative to their state, efficiently binding the effectors together to achieve well-timed coordination. Indeed, it would be detrimental to actions such as throwing a ball to a target to time arm and wrist movements based on independent time estimates. Independent noise levels or drifts would quickly lead to a decoupled motor state where the timing of muscle activations is disrupted, as in cerebellar ataxia, and may lead to a state resembling movement decomposition (Bastian et al. 1996; Timmann et al. 1999).

The impact of overlap between different motor activity states on their temporal encoding echoes the findings on discrete (non-overlapping) versus continuous (overlapping) timing tasks. Ivry and colleagues suggested a dichotomy of dedicated versus emergent encoding of timing for discrete versus continuous movements, respectively (Spencer et al. 2003; Ivry and Spencer 2004; Ivry and Schlerf 2008). Temporal variability on continuous tasks characterized by smooth transitions between different motor states (e.g. circle drawing) have been reported to be uncorrelated with the temporal variability on discrete tasks characterized by movement pauses in between boosts of motor activity (e.g. tapping) (Zelaznik et al. 2005). Moreover adjustment to timing perturbations is faster and more precise for discrete as opposed to continuous movements (Elliott et al. 2009; Repp and Steinman 2010; Studenka and Zelaznik 2011) and patient studies suggest that these movements might rely on different neural substrates (Spencer et al. 2003; Spencer and Ivry 2005). Yet, it is unlikely that movement kinematics alone determine whether temporal encoding is dedicated versus emergent: As discussed above, even continuous movements like smooth pursuit can be controlled using dedicated timing mechanisms and independently of parameters such as movement velocity, whenever the absolute timing predicts task success (Medina et al. 2005), or when a periodic circle drawing tasks contains a salient auditory cue marking the completion of a cycle (Zelaznik and Rosenbaum 2010; Braun Janzen et al. 2014).

When it comes to dissociating the spatial and temporal organization of sequential motor skills, the focus has been on learning the organization of sequences of movements rather than on learning the production of the constituent movements per se. Thus, typically subjects are trained to sequence simple overlearned movements like finger presses (Sakai et al. 2003; Ullen and Bengtsson 2003; O’Reilly et al. 2008; Kornysheva et al. 2013; Kornysheva and Diedrichsen 2014). With training the production of sequences becomes more accurate and is retrieved faster as evidenced by shorter sequence duration or reaction times (RT) depending on the task employed. In addition, a temporal grouping idiosyncratic to the subject or facilitated externally by the sequence structure emerges, such that certain movements in the sequence become closer in time than others creating so-called chunks. There is compelling evidence that breaking up the sequence within chunks as opposed to between chunks when reordering the sequence leads to losses in performance [for reviews see (Sakai et al. 2004)]. This suggests that a dedicated representation has been formed for each chunk of movements in space which facilitates performance—similar to chunks in working memory and cognitive control (Baddeley 2010). It has been hypothesized that this temporal grouping is a sign of a skill becoming automatic and pairing the sequence with a different temporal structure would lead to losses in performance as this automatic representation has not been formed (Hikosaka et al. 2002; Sakai et al. 2004).

Interestingly, there is evidence that while changing a chunking structure (externally induced) can lead to performance losses, these are not as pronounced as when performing a novel sequence (O’Reilly et al. 2008). This suggests some form of independence for the spatial organization of sequences, on top of the integrated spatio-temporal chunking structure. In contrast, many studies have shown that retaining the timing while changing the spatial feature of movement sequences does not provide any benefit as compared to a new sequence, which advocates that the temporal structure of these sequences is invariably bound to their sequential movements in space (Shin and Ivry 2002, 2003; O’Reilly et al. 2008).

**Fig. 2:**
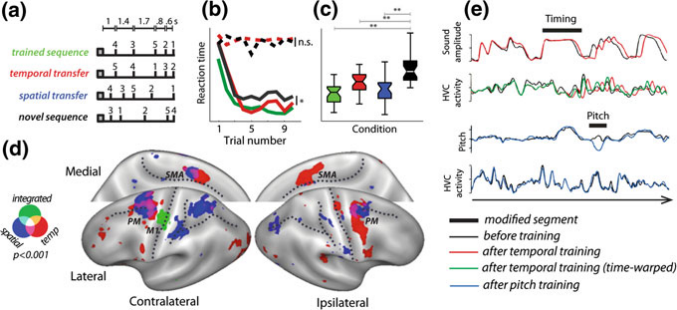
Evidence suggesting that spatial and temporal features of movement sequences are represented independently. **a** Participants were trained on a specific spatio-temporal finger sequence (*green*) and then tested on a novel sequence (*black*) or on sequences that retained either the temporal (*red*) or spatial (*blue*) structure (Kornysheva et al. 2013; Kornysheva and Diedrichsen 2014). The numbers 1–5 in exemplary sequences correspond to the thumb, index, middle, ring and little finger, respectively. **b** Reaction time advantages relative to a new sequence that are related to a learned trained temporal feature can only be expressed when the spatial feature becomes more predictable. Solid lines correspond to “trained”, “temporal” and “novel” conditions in which the corresponding sequences are presented 10 times in a row, whereas the dashed lines correspond to conditions where the trained temporal feature is paired with a new spatial feature on every trial (*dashed red*) and compared to a sequence that changes both the temporal and the spatial feature on every trial. Stars indicate significant differences across trials (Kornysheva et al. 2013). **c** Reaction time results indicate independent transfer of spatial and temporal features to test conditions (Kornysheva and Diedrichsen 2014). **d** Separate, but partly overlapping spatial (*blue*) and temporal (*red*) representations of finger sequences can be revealed bilaterally in premotor cortex (PM and SMA) using multi-voxel pattern analysis. The two features are integrated in contralateral M1 only (*green*). In a series of behavioural and fMRI experiments employing (Kornysheva and Diedrichsen 2014) **e** The premotor nucleus HVC in zebra finches reflects changes in the temporal feature of a bird song (*red line*), such as a prolonged syllable, but not changes in its pitch feature (*blue line*). Both types of changes were acquired through aversive conditional auditory feedback (adapted from Ali et al. 2013)

This, however, has been challenged recently in a series of experiments (Kornysheva et al. 2013; Kornysheva and Diedrichsen 2014). Here the experimental test involved producing sequences following training of a single spatio-temporal sequence of finger presses in a timed SRT task (Penhune and Steele 2012). These were either repeated in a block of several trials or new on every trial. The results suggested that RT savings for a trained temporal feature paired with a new sequence of finger presses (spatial feature) could only emerge once the new spatial feature became more predictable through repetition (Fig. 2a, b). Note that the advantage for the trained temporal features is relative to the control condition in which the sequence was also repeated and the finger sequence became equally more predictable with repetition. In contrast when the finger order was new on each trial comparable to the random spatial sequence controls in the studies discussed above, there was no advantage related to learning the timing of the sequence. It is unlikely that this is an effect of whether these sequences were learned implicitly or had an explicit component, as both the presence and the absence of temporal transfer were found depending on the familiarity with the spatial feature.

More formally, drift diffusion modelling demonstrated that these results can be best approximated using a multiplicative integration of independent spatial and temporal sequence feature representations as follows such as *Z*_*n*+1_ = *Z*_*n*_ + *V* + *S* + (*S* * *T*), rather then an additive integration (*Z*_*n*+1_ = *Z*_*n*_ + *V* + *S* + *T*), or a combined spatio-temporal term without a separate temporal representation (*Z*_*n*+1_ = *Z*_*n*_ + *V* + *S* + *C*). Here *Z* is the selection layer corresponding to the five fingers, *V* is the visual stimulus in the serial reaction time task (SRTT), *S* the spatial, *T* the temporal and *C* the combined representation in which the temporal sequence feature is linked to a specific spatial feature (weights and noise terms are omitted for abbreviation purposes). Essentially, this means that while effects of the spatial feature representation act independently (additive integration) the temporal representation can only be expressed when S > 0, in other words there is some knowledge of the spatial representation. The difference between an integrated spatio-temporal versus an independent temporal representation which is multiplicatively combined with the spatial one is critical, as only the latter allows for temporal transfer which we could reliably observe across experiments (Fig. 2b, c).

A subsequent study investigated how independent and integrated spatial and temporal representations are represented across the neocortex and the cerebellum based on fine-grained local fMRI activity patterns (Kornysheva and Diedrichsen 2014). Despite the low resolution (fMRI voxels) these neural representations can be probed due to tiny, but systematic spatial activity biases which occur with trial repetition. Here instead of training one particular spatio-temporal sequence, subjects were trained to produce nine spatio-temporal finger sequences, which were unique combinations of three finger order (spatial feature) and temporal interval (temporal feature) sequences. This factorial design in combination with multivariate pattern analysis allowed to test for local voxel activity patterns related to the spatial feature across sequences with different temporal features, and orthogonally, patterns related to the temporal feature across different spatial features—feature transfer on the neural level. Moreover, subtracting out the main effects of independent spatial and temporal features from the overall activity patterns isolated residual patterns, which, if unique for each sequence, were taken as integrated neural representations.

The results revealed that fine-grained patterns in overlapping patches of the lateral (dorsal and ventral) and medial (SMA) premotor cortex carried information on the independent spatial as well as independent temporal patterns, whilst the only region informative of an integrated spatio-temporal representation was the contralateral primary motor cortex, the output stage of the neocortex (Fig. 2d). Thus, in M1 each sequence may recruit a subpopulation of neurons that controls a particular combination of spatiotemporal synergies (d’Avella et al. 2003). The latter cannot be synergies of individual finger movements as each finger movement occurred in each sequence, but particular spatio-temporal transitions within sequences. The same principle, but now for spatial and temporal parameters would apply for the premotor cortex—unique combinations of synergies capturing particular spatial (timing-invariant) or temporal transitions.

The alternative is that the encoding observed in M1 is not sequential encoding per se, but reflects the two spatial and temporal codes being combined nonlinearly. Also while the force level for each finger matched well across sequences, it cannot be completely excluded that tiny biases—thumb, index finger, etc., being more active in one sequence than in another—may have contributed to the encoding to some extent. Yet, this explanation is unlikely, since encoding in contralateral M1 correlated with sequence learning, but not with sequence classification accuracy based on the force at each finger.

The presence of independent spatial and temporal codes, as well as integrated representations suggests varied levels of abstraction from the actual motor response implementation. To be transferable across different temporal profiles, the spatial sequence in the premotor cortices has to lack specifics on the kinematics or dynamics of each effector involved during sequence production, and may carry more abstract information such as on sequential transitions between movements (Tanji and Shima 1994). Conversely, the temporal feature representation is bound to lack any information on the effectors and the dynamics such as force on each finger to be transferable across different finger movement sequences.

Interestingly, a similar dissociation in the control of spatial (pitch) and temporal sequences has been found in songbirds (Ali et al. 2013). Using aversive auditory conditioning, the authors taught the animals to selectively modify temporal and spectral features of their song, such as changing the length of a syllable, or its pitch which requires a different configuration of muscle activations controlling the syrinx (Fig. 2e). The basal ganglia analog was required for the modification of the spectral properties (pitch), but not for changes in the temporal structure. By contrast, the activity in HVC (an analog to the premotor cortex) reflected the temporal but not spectral features of the song. This dissociation and therefore modularity of spatial and temporal features in motor sequence control may thus be a universal property of the CNS.

These findings resonate with the hypothesis by d’Avella and colleagues suggesting that the control of movement may be modular during a variety of reaching movements (d’Avella 2017), as the variability of muscle activations recorded as EMG signals can be explained by three types of components, so called muscle synergies: (a) time-invariant spatial (S), (b) muscle-invariant temporal (T), (c) as well as muscle-specific spatio-temporal synergies (ST). S are the activation weights on each muscle required for the movement, which do not specify any change over time, T are the temporal activation profiles which are shared across different muscles and ST are activation waveforms for specific muscles which amount to an idiosyncratic dynamical trajectory of individual muscles. These results suggest that at the muscular level the underlying temporal features of movements are transferable across different muscle synergies, respectively. Although explaining variability of muscle activations by synergies does not provide direct evidence for the encoding of these synergies in the CNS, these results allow for the possibility of controllers somewhere in the corticoid-spinal pathway that impose this modular regularity on motor output. A recent analysis of premotor and primary motor units provided the first evidence that neural activity in the CNS can be explained by EMG synergies (Overduin et al. 2015).

A modular representation enables a radical simplification of motor control policies: Instead of controlling the spatio-temporal evolution of each individual muscle throughout the movement, the CNS triggers spatial and temporal synergies required for the skilled movement. Moreover, instead of encoding all combinations of movements, the brain utilizes temporal and spatial synergies or profiles which can be recombined flexibly into different combinations. If skilled movements did not in principle require a dedicated representation of their temporal dimension and were merely emergent from the encoding of the dynamics of the movement they are performed with, such learned movements would be rigid with regard to their temporal evolution beyond a simple speed up of slow down. It would entail that the temporal dimension could not be utilized across different effectors and motor states. Coming back to the musical example, the violinist would have to form an entirely new representation whenever the temporal structure of a sequence is modified or whenever a new sequence of movements is paired with a familiar temporal structure, which contradicts the findings above.

## Computational Models and Neural Mechanisms of Temporal Representation (“How”)

It has been hypothesized that a variety of neural structures are capable of encoding the timing of movements, which corresponds to the widespread involvement of these areas in explicit or implicit motor timing tasks—in particular the cerebellum, the striatum and the lateral and medial premotor cortices (Lewis and Miall 2003; Buhusi and Meck 2005; Ivry and Schlerf 2008; Buonomano and Laje 2010; Teki et al. 2011; Laje and Buonomano 2013). This is surprising as these different parts of the nervous system have diverse neural architectures, as well as physiological and computational constraints. Conversely, such diversity suggests that these systems are unlikely to be redundant with respect to skilled motor timing, specializing on a particular neural computation which determines or co-varies with motor timing. Below I will present a hypothesis of how such parallel processes may operate and interact to enable precise motor timing based on results from computational modelling and current neuroscientific evidence.

The cerebellar cortex has been one of the first regions hypothesized in motor and more generally sub-seconds timing (Braitenberg 1967). In stark contrast to the neocortex, the architecture of the cerebellar circuitry is remarkably uniform across the different parts of the cerebellum (with the exception of the floccular cortex) with the main difference between regions being the origins of their inputs and the targets of their outputs. The circuitry is designed to integrate only two types of inputs from the rest of the nervous system, which converge in the cerebellum: The mossy fibre pathway that relays information from the cortex (via the pons), as well as the periphery (via the brainstem) and the climbing fibre pathway that carries signals from the inferior olive in the brainstem. The cerebellar output is sent to the neocortex via the thalamus or to the periphery via brainstem nuclei, and has been shown to form reciprocal multisynaptic cortico-cerebellar loops (Kelly and Strick 2003).

While the deep cerebellar nuclei (DCN) receive excitatory input *directly* via mossy and climbing fibre collaterals, the anatomical connections of the two fibre systems to the Purkinje cell (PC) layer is at the core of cerebellar architecture: Unlike to the DCN, the mossy fibre to PC projection is *indirect*, going through a layer of granule cells, which remarkably constitute the majority of neurons in the brain. Granule cells relay this information by parallel fibres that run transversally through flattened and orthogonally oriented dendritic trees of PCs with some of which they form direct excitatory connection on the way, and inhibit them indirectly via the inhibitory interneurons. Remarkably, Purkinje cells have a baseline firing rate of 50–100, sometimes up to 200 Hz (Zeeuw et al. 2011; Zhou et al. 2014), and inhibitory projections to the DCN as their only output (GABA). They act as a constant break on the DCN, which activity is released only when the PCs exhibit a firing pause that in turn disinhibits the DCN, the sole output of the cerebellum.

The granular layer has been hypothesized to act like a giant “filter” of the mossy fibre input (Dean et al. 2009, 2013) redistributing the mossy fibre inputs across granule cells (divergence), but at the same time mixing inputs from different channels—sensory and motor at the single cell level (Huang et al. 2013; Ishikawa et al. 2015). In classical eyeblink conditioning, which acts as a model for the learning of timed motor responses, time varying activity in a subset of granule cells activated by the conditioning stimulus (CS) has been hypothesized to produce a temporal code at the parallel fibre to PC synapses (Medina and Mauk 2000). This synaptic input to the PC can act as a clock, as each unique state of the synaptic input after a stimulus corresponds to the passage of time following the CS onset. In contrast, learning of the precisely time motor response (eyeblink) takes place based on an aversive stimulus, such as a short air-puff directed into the eye (unconditioned stimulus, US). The latter is transmitted by the climbing fibre system, and leads to the depression of those parallel fibre to PC synapses active just before the time of the aversive stimulus, partly mediated by plasticity in interneurons inhibiting the PC (Medina and Mauk 2000; Heiney et al. 2014). This eventually leads to decreased PC simple spike cell firing during the interval between the two stimuli with the most pronounced reduction timed just before the conditioned response (CR), the latter being initiated via the disinhibition of the DCN (Jirenhed et al. 2007; Ten Brinke et al. 2015). It has been repeatedly shown that the intact cerebellar cortex is necessary for a precisely timed response, as the intact DCN alone produces a short-latency response without any temporal features necessary for the task (Perrett et al. 1993; Koekkoek et al. 2003). Importantly, this notion advocates a distributed motor learning architecture across the cerebellum (Gao et al. 2012), and argues for a special role of the cerebellar cortex in motor timing.

More recently it has been proposed that the temporal profile of the response can be acquired locally in the PC (Johansson et al. 2014). Specifically, pairing a CS consisting of a direct stimulation of the parallel fibres (circumventing the granular cell layer) with a US consisting of direct climbing fibre stimulation led to a Purkinje cell CR that was adaptively timed. The cell reached maximum suppression of 75 ms before the onset of the US across different CS-US intervals. Importantly, even when blocking inhibition from inhibitory interneurons that are also innervated by parallel fibres and could have had an effect on the PC response, the learned timing was preserved. This led the authors to conclude that the encoding of the precisely timed response is located in the PC at the molecular level. Specifically, blocking mGluR7 receptor has been shown to disrupt timing in the direct stimulation paradigm above (Johansson et al. 2015). While the exact mechanism of molecular timing is still unknown, it has been hypothesized that the CS may initiate a predictable biochemical cascade while the US onset induces interval-specific changes to this cascade. This could take place in form of a selection of different molecular components with particular properties with regard to the duration of ion channel open states, so that the time course of the PC simple spike depression matches the CS-US interval.

Regardless of whether the timing mechanism is distributed or localized, the parts of the cerebellar cortex involved in classical conditioning project to a specific target effector in the periphery and cannot be expected to be transferable across different effectors, spatial configurations or motor states. For instance, the cerebellar cortical projection to the anterior interpositus of the DCN nucleus involved in eyeblink conditioning innervates periorbital muscles of the eye via the brain stem (Ten Brinke et al. 2015). However, a more abstract representation of timing for spatio-temporal movements is still conceivable in those regions of the cerebellum that project to the premotor and prefrontal cortices via the dentate nucleus (Kelly and Strick 2003), albeit only if they receive climbing fibre stimulation at the time of the US during learning which has not been investigated systematically so far.

Another timing mechanism has been attributed to the basal ganglia, the striatal beat frequency model (Matell et al. 2004; Buhusi and Meck 2005). Unlike the cerebellar timing mechanisms described in this chapter, the latter is relevant for interval timing that involves intervals of seconds-to-minutes. While even the lower range may appear too long to be relevant for motor timing many skilled movements like verbal utterances, musical and dance sequences, as well as the typing Morse code messages involve sequences of movements that unfold over the timescale of several seconds to tens of seconds. The basal ganglia is organized in cortico-basal ganglia-thalamo-cortical loops with the majority of the excitatory input coming from the cortex and then sent out to direct and indirect pathways of the basal ganglia which excite and inhibit the cortex, respectively, via the thalamus (Graybiel 1998). Here each medium spiny neuron in the striatum receives up to 30.000 separate axons from the cortex. Thus, it has been proposed that through learning the medium spiny neurons in the striatum act as coincidence detectors of neural oscillations that operate at different frequencies in the neocortex (Buhusi and Meck 2005). With trial onset the phase of the oscillations is reset (“start-gun”). During learning a reward signal at the end of the interval to be trained is conveyed by dopaminergic input from the substantia nigra pars compacta and the ventral tegmental area. Experience-dependent changes in cortico-striatal transmission (both LTP and LTD) lead to a ramp of striatal activity with a peak at the time of the expected reward, i.e. at the end of the interval. Accordingly, following training striatal neurons may be capable of detecting the unique coincidence of phases of the neural oscillators that project to these neurons, respectively. Interestingly such adaptively timed ramping activity has also been observed in the neocortex, such as in a motor synchronization-continuation task involving isochronous intervals performed at different speeds in the monkey supplementary motor area (SMA) (Merchant et al. 2013) and an interval reproduction task in the parietal cortex (Jazayeri and Shadlen 2015). Although there has been no direct experimental evidence from studies involving sub-second intervals, it is likely that such ramps reflect the striatal activity via the direct basal ganglia thalamic route to the neocortex. Indeed, imaging, lesion and pharmacological studies have confirmed the involvement of the striatum in interval timing (for a review cf. Buhusi and Meck 2005).

Finally, the neocortex could be regarded as most closely related to models involving random recurrent networks (Thomson and Bannister 2003; Buonomano and Laje 2010). Recent concurrent multiunit recordings from premotor and primary motor cortices suggest that the trajectory of a movement is not represented in terms of its features such as position, velocity, direction, force and timing as suggested before, but rather as a compound of variables correlated leading to the performed trajectory in space (Churchland et al. 2006; Shenoy et al. 2012; Kaufman et al. 2015). Here the timing is merely an emergent feature of the evolution of the multiunit activity which controls the spatial movement trajectory. Accordingly, a model of randomly connected networks can be trained to produce skilled sequential movements and have perfectly reproducible temporal dynamics without any dedicated encoding of the temporal dimension in the model (Laje and Buonomano 2013). Such a network of interconnected units can be trained to represent the spatio-temporal evolution of a trajectory as complex as handwriting (in two-dimensional space).

Central to the function of this model is a random recurrent network of interconnected firing-rate nodes with a multiunit firing rate that learn to follow a particular innate trajectory depending on the input trigger. Learning consists of the reduction of the variability in this innate trajectory in space by adjusting the network weights enabling the firing rate activity to be robust to noise and perturbations, so that the trajectory can return to a carved out path. This network activity can be read out continuously by an output module that maps its multiunit state into external variables like an x and y position for complex motor trajectories and could in principle also guide movements in muscle space. The timing of this movement is also reliable after training, such that a certain position in space clusters equally tightly in time. This is despite the temporal features of the movement not having a dedicated representation, but emerging from the dynamics of the trajectory dedicated to the spatial position of the movement.

While the dynamical systems view focuses on the representation of a movement in space with timing being an emergent property of the trajectory, Buonomano and colleagues proposed that the dynamical trajectories produced by random recurrent networks could also be utilized to encode discrete timing of movements (Buonomano and Laje 2010). These networks could be trained to control a simple timing task, producing a phasic pulse after a specific interval (activity in one-dimensional space y), analogous to a discrete button press in a finger tapping task or eyelid closure in eyeblink conditioning. Computationally the mechanisms of such dedicated temporal representations are equivalent to the encoding of the continuous spatio-temporal trajectory. What is crucial here is the mapping of the network output to a readout unit controlling a motor response. This mapping determines whether the timing is a by-product of the spatial trajectory or whether the network activity which is consolidated after training essentially acts as a population clock, triggering a discrete response once the network activity reaches a particular state. The latter can be extrapolated to sequential representations of finger movement sequences. Thus, from the perspective of the neocortex discrete event timing and continuous emergent timing which have been tied to distinct neural substrates as discussed earlier (cf. Spencer et al. 2003) could in principle be encoded in the same way.

This flexibility of temporal encoding in the networks resembling the neocortex resonates with the imaging results showing independent temporal and spatial feature encoding in the premotor cortices versus integrated spatio-temporal encoding in contralateral primary motor cortex (Kornysheva and Diedrichsen 2014; Diedrichsen and Kornysheva 2015). Within the dynamical systems framework, this modularity would be related to the activation of several recurrent neocortical networks that are utilized to encode integrated spatio-temporal encoding in M1 and dedicated temporal encoding in premotor regions, the latter enabling the flexibility of the response independently of a spatial motor features, analogous to the temporal transfer observed behaviourally (Fig. 2a–d). In contrast, it is much less straightforward how such recurrent networks could be mapped to encode the spatial feature of sequences (e.g. finger order) independently of their exact temporal feature. If the encoding of movement sequences draws on consolidated multiunit trajectories of randomly recurrent units, the precise changes in multiunit space would be ignored, such that a certain cascade of states would be mapped onto the same spatial state (configuration of finger activations). The temporal evolution would then be specified at the stage when both are combined either by acting on integrated spatio-temporal M1 representations (Kornysheva and Diedrichsen 2014) or downstream in the case of direct cortico-spinal projections from the premotor cortex.

How do these regions interact with each other to achieve precise motor timing of skilled movements? Here only projections with a short latency (“online”) transduction up to tens of milliseconds can be considered to exhibit control at time scales relevant to online motor control.

For a long time it has been assumed that the basal ganglia and the cerebellum operate in parallel to each other at the subcortical level, having separate thalamic relays to the neocortex (Bostan et al. 2013). However, in rodents (Ichinohe et al. 2000) and more recently in primates (Hoshi et al. 2005; Bostan et al. 2010) disynaptic connections from the DCN to the striatum have been established. The relay is located in the intralaminar nuclei of the thalamus which contain projections to the striatum. Recently, it has been determined that the propagation speed between DCN and the dorsolateral striatum can be as low as 10 ms (Chen et al. 2014). This suggests a rapid transmission of cerebellar output to striatal. High frequency and well-timed bursts in DCN neurons can modulate activity at the entry stage of the basal ganglia, thereby coordinating cerebellar output with the basal ganglia computations in real time. Interestingly when stimulation of the DCN was combined with concurrent cortico-striatal input, the cortico-striatal activation was potentiated (Chen et al. 2014). The cerebellar output signals which carry a high temporal resolution profile of a signal are therefore impacting the neocortical input at the level of the ramping activity of medium spiny neurons. At the same time the subthalamic nucleus to which striatal neurons project via the indirect pathway innervates the cerebellum via the pontine nuclei. The propagation speed of this connection is currently unknown.

As with the basal ganglia, the premotor cortex forms reciprocal disynaptic connections with the cerebellum (Kelly and Strick 2003). The DCN project to the neocortex via the ventrolateral nucleus of the thalamus and affect not only supragranular layers, but also directly layer V in M1 as shown by optogenetic stimulation of the cerebellar Purkinje cells (Proville et al. 2014). Importantly, the DCN inhibition is followed by a rebound excitation following the offset of Purkinje cell stimulation at around 60ms and in M1 40ms later. At the same time this study revealed a short-latency transmission between M1/S1 and the lateral cerebellar cortex, with onsets of Purkinje cell frequency modulation as early as 10ms after neocortical stimulation. Finally, non-invasive research in humans has shown that the latency of cerebellar inhibition of the cortex as measured by M1 triggered MEP is highest at 5 ms delay (Ugawa et al. 1991), confirming a rapid transmission between the cerebellum and the neocortex. In other words, it is likely that the (pre-)motor cortical networks relevant for temporal encoding receive a precisely timed (high resolution) signal from the cerebellum while the latter is modulated by neocortical input, with these interactions unfolding almost instantaneously.

**Fig. 3:**
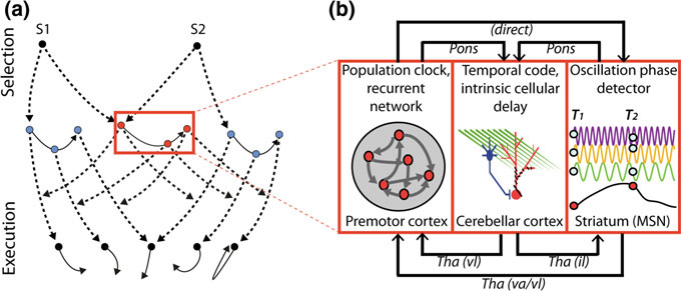
Temporal encoding for skilled spatiotemporal sequence production. **a** Modular representation of temporal (*red dots*, a longer and a shorter interval) and spatial (*blue dots*) sequence features. The temporal representation modulates the signal originating from two different spatial representations (*black broken arrows*) (Kornysheva et al. 2013). This allows two different sequences S1 and S2 to utilize the same learned temporal structure flexibly (adapted from Diedrichsen and Kornysheva 2015). **b** The premotor cortex, the cerebellar cortex and the striatum utilize different computational mechanisms that can be harnessed to learn and control motor timing—either independently of the movement in space as shown here or in an integrated spatio-temporal fashion (see main text). These regions are interconnected with each other by short-latency circuits via the thalamus and the pons, respectively. The following model of motor timing for skilled movement sequences is proposed in the current review: The neocortex produces sustained dynamic activity in a population of interconnected neurons which can be utilized for the duration of a whole sequence of movements (Buonomano and Laje 2010). This multi-unit activity is read out by the MSN in the striatum based on oscillation phase detection and chunked into a series of ramps that mark the interval between movement onsets or between an external stimulus and a motor response (Buhusi and Meck 2005). Crucially, the cortical and striatal activity is fed into the cerebellum, providing a sequential context signal for each movement unfolding in the seconds time range. This activity is transformed by the cerebellar cortex into a precise high temporal resolution output on a sub-seconds scale in the deep cerebellar nuclei for each sequence component. Through disynaptic projections, the latter modulates both the ramps in the striatum and the population clocks in the neocortex to achieve a more precisely timed representation of the sequence. Abbreviations: il—intralaminar; MSN—medium spiny neurons; S—sequence; T—time point; Tha—Thalamus; va—ventroanterior; vl—ventrolateral

Why do we need parallel timers in our brain operating in parallel and what is their specific contribution? A schematic model based on the current review is presented in Fig. 3 (cf. caption for details). At the current stage, any answers to this question will remain speculative. Most of the invasive electrophysiological recordings that could provide direct evidence for this report only from one region at a time. Yet, in an intact brain it is impossible to disentangle whether the activities reported relay the input of interconnected regions, or whether this activity originates and is causally involved in the production of well-timed movements. Even lesion studies (temporal inactivation, TMS, patients, etc.) are of limited use, as they cause reorganization in the network, that unless recorded, remains hidden and may impact conclusions with regard to behaviour. Short-lived local inactivation though muscimol, optogenetic stimulation (animal models) and transcranial magnetic stimulation (humans) whilst recording from the site to which the region that is disrupted projects are likely to provide more conclusive answers to this question. For instance, to assess the individual contribution of cortical and subcortical sites to learned timing, a pioneering study by Mauk and colleagues has been conducted to decompose the contributions of the neocortex versus the cerebellar nuclei to trace eye blink conditioning (Siegel and Mauk 2013). This task is known to rely not only on the cerebellum (in contrast to delay eyeblink conditioning), but also on the cortex and the hippocampus. Here it could be demonstrated that ramping activity observed in prefrontal cells, as well as the well-timed conditioned motor response is abolished when cerebellar output is inhibited, whereas the sustained activity during the duration of the CS remained intact. In the future similar studies need to be designed to directly probe the contribution of the premotor cortex, the striatum and the cerebellar cortex to skilled motor timing.

## Conclusions/Take Home Message

Precise motor timing of spatio-temporal skills is crucial for a variety of skilled movements. During the past decade there have been contradictory results with regard to how timing for spatio-temporal motor skills is represented in the brain. The encoding of motor timing is achieved either directly by measuring time intervals from movement onset or an external stimulus (dedicated timing) or indirectly via state-dependent encoding (emergent timing). Which mode is chosen depends on the characteristics of the motor task, such as the correlation of the temporal target with a state-dependent variable (e.g. position or velocity), the presence of temporal overlap across effectors requiring their coordination in time and the reliability of temporal versus state-dependent encoding for task success. The ability to transfer temporal features across different motor configurations in space indicate a modular representations of these features for the control of skilled motor sequences which can be found in the premotor as opposed to primary motor cortices. The idea that there is a localizable universal neural clock in the CNS, which is utilized across different domains, perceptual and motor, is an unlikely scenario. Partly this is evidenced by the fact that timing functions have been attributed to different areas across the brain. Instead, different neural mechanisms that operate in parallel—dynamical systems (random recurrent network), oscillation phase detection (ramps), patterned input and molecular delays at the cell level—constitute representations in neocortical motor areas, the striatum and the cerebellar cortex, respectively. These neural representations interact with each other in short-latency loops to produce well-timed behaviour.
